# Supplementary material for: Multi-scale computer-aided design and photo-controlled macromolecular synthesis boosting uranium harvesting from seawater
Source: Nat Commun. 2022 Jul 7;13:3918. doi: 10.1038/s41467-022-31360-x (PMC9262957; doi:10.1038/s41467-022-31360-x)
Supplement: Supplementary file 1 — Supplementary Information [file 41467_2022_31360_MOESM1_ESM.pdf]

Supplementary Information

Multi-scale computer-aided design and photo-controlled macromolecular synthesis boosting uranium harvesting from seawater

Zeyu Liu[1,⊥], Youshi Lan[2,⊥]Jianfeng Jia[1], Yiyun Geng[1], Xiaobin Dai[3], Litang Yan[3], Tongyang Hu[1], Jing Chen[1], Krzysztof Matyjaszewski[4,], and Gang Ye[1,*]*

[1] Collaborative Innovation Center of Advanced Nuclear Energy Technology, Institute of Nuclear and New Energy Technology, Tsinghua University, Beijing 100084, People's Republic of China

[2] China Institute of Atomic Energy, Department of Radiochemistry, Beijing 102413, People's Republic of China

[3] State Key Laboratory of Chemical Engineering, Department of Chemical Engineering, Tsinghua University, Beijing 100084, People's Republic of China

[4] Department of Chemistry, Carnegie Mellon University, 4400 Fifth Avenue, Pittsburgh, Pennsylvania 15213, United States

[⊥] These authors contributed equally

Contents

**Supplementary Derivations**	3
**Supplementary Experimental Details**	5
**Supplementary Figure 1**. Schematic illustration of conceptual polymer chains with fully stretched conformation (a) and completely collapsed conformation (b).	8
**Supplementary Figure 2.** A general flowchart implemented in the computational simulation of uranium adsorption by AO-functionalized polymeric adsorbents.	9
**Supplementary Figure 3. Variation of $R_{g,PAO}$ with running steps in computational simulation. a** PAO_{40}-*b*-$PPEGMA_{10}$ with different DCFs. **b** PAO_{40}-*b*-$PPEGMA_{20}$ with different DCFs. **c** PAO_{40}-*b*-$PPEGMA_{30}$ with different DCFs. **d** PAO_{40}-*b*-$PPEGMA_{40}$ with different DCFs. **e** PAO_{40}-*b*-$PPEGMA_{50}$ with different DCFs.	10
**Supplementary Figure 4.** Molecular weight distribution (MWD) of the PAN-*b*-PPEGMA obtained in the PET-RAFT polymerization presented as the number distribution.	11
**Supplementary Figure 5.** GPC profile of the PAN-CTA synthesized in this study.	12
**Supplementary Figure 6. ^{1}H NMR spectra of PAN and PAN_m-*b*-$PPEGMA_n$ with different block ratios. a** Homopolymer PAN. **b** PAN_m-*b*-$PPEGMA_{n1}$. **c** PAN_m-*b*-$PPEGMA_{n2}$. **d** PAN_m-*b*-$PPEGMA_{n3}$. **e** PAN_m-*b*-$PPEGMA_{n4}$.	13
**Supplementary Figure 7. FT-IR spectra of homopolymer PAN and the block copolymers**. **a** PAN. **b** PAN_m-*b*-$PPEGMA_n$. **c** PAO_m-*b*-$PPEGMA_n$.	14
**Supplementary Figure 8.** Contact angle measurements of the electrospun nanofibrous membranes of pristine PAO and PAO_m-*b*-$PPEGMA_n$ after uranium adsorption.	15
**Supplementary Figure 9.** Adsorption of uranium in real seawater by PAO_m-*b*-$PPEGMA_n$ with a block ratio n/m of 0.17.	16
**Supplementary Figure 10.** SEM image of the regenerated ENM of PAO_m-*b*-$PPEGMA_{n3}$ after uranium elution.	17
**Supplementary Table 1.** The linear fitting of the relationship between the equilibrium $R_{g,PAO}$ of PAO_m-*b*-$PPEGMA_n$ with different block ratio n/m and the dead chain fraction (DCF, %) in their structures, $R_{g,PAO} = a \times DCF + b$..	18
**Supplementary Table 2.** The parameters of ΔE_{ij}^{mix} used in the computational study.	19
**Supplementary Table 3.** The parameters of χ_{ij} used in the computational study.	20
**Supplementary Table 4.** Repulsion parameters α_{ij} used in computational study.	21
**Supplementary Table 5.** The adsorption capacity and kinetic fitting parameters of the ENMs of PAO_m-*b*-$PPEGMA_n$ in uranium-spiked water.	22

Supplementary Derivations

Derivation S1

As shown in Figure S1a, the polymer chain looked like a string, so the center of gravity of the molecule is at the middle side of the chain, and the polymer conformation is completely symmetrical, so we can get equation S1:

$$R_{\mathrm{g}}^2=\frac{\sum m_{\mathrm{i}} r_{\mathrm{i}}^2}{\sum m_{\mathrm{i}}}=\frac{L^2+(2L)^2+(3L)^2+\cdots+(NL/2)^2}{N/2}=\frac{1^2+2^2+3^2+\cdots+(N/2)^2}{N/2}\times L^2\approx\frac{N^2}{12}L^2 \quad \text{(S1)}$$

Under this condition, all of the binding sites are accessible to the uranyl ions. Supposing that the accessible functional groups coordinate to uranyl ions according to a 2:1 molar ratio, a classical coordination mode between AO ligands and uranyl carbonates in seawater, the maximum theoretical adsorption capacity ($\Gamma_{\max}$) can be obtained as:

$$\Gamma_{\max}=\frac{N}{2} \quad \text{(S2)}$$

where m_{i} is the mass of the ith part of monomer; r_{i} is the distance between the center of gravity of the ith part of monomer and the center of gravity of the whole polymer chain; N is the degree of polymerization; L is the hydrodynamic diameter of single monomer.

Derivation S2

As shown in Figure S1b, when the polymer chain was completely folded with a fully condensed conformation. To simplify the calculation, a monomer unit is considered to a cube with a length equal to L, and a big cube with the same size as the hydrodynamic diameter of the polymer chain to was employed to substitute the sphere during the following derivation. We can get the side length (a) of this cube, $a=\sqrt[3]{N}L$, and the center of gravity of the polymer chain at the body center of the cube. So, the following equation was obtained to express the R_{g}^2 :

$$R_{\mathrm{g}}^2=\frac{\sum mr^2}{\sum m}=\frac{1}{N}\sum_{\mathrm{i,j,k}=0}^{\mathrm{i,j,k}=\frac{\sqrt[3]{N}}{2}}(\mathrm{i}L)^2+(\mathrm{j}L)^2+(\mathrm{k}L)^2$$

$$=\sum_{\mathrm{i,j,k}=0}^{\mathrm{i,j,k}=\frac{\sqrt[3]{N}}{2}}(\mathrm{i}^2+\mathrm{j}^2+\mathrm{k}^2)L^2$$

$$=\frac{(\sqrt[3]{N}+1)(\sqrt[3]{N}+2)}{4}L^2\approx\frac{N^{2/3}}{4}L^2 \quad \text{(S3)}$$

And the binding sites can be calculated by the specific surface area:

$$\Gamma_{\max(\mathrm{cube})}=\left[6\times\left(\sqrt[3]{N}L\right)\times\left(\sqrt[3]{N}L\right)\right]\div 2L^2=3N^{2/3} \quad \text{(S4)}$$

For the sphere which has the same volume as the cube, the radius (r) can be calculated as:

$$a^3=\frac{4}{3}\pi r^3\Longrightarrow r=(\frac{3}{4\pi})^{1/3}a \quad \text{(S5)}$$

So, we can get the ratio between specific surface area of the sphere and that of the big cube:

$$\alpha=\frac{6a^2}{4\pi r^2}=(\frac{1}{6\pi^2})^{1/3} \quad \text{(S6)}$$

Bring equation S6 into equation S4, we can get the amounts of binding sites:

$$\Gamma_{\max}=\alpha\times\Gamma_{\max(\mathrm{cube})}=(\frac{1}{6\pi^2})^{1/3}\times 3N^{2/3}=(\frac{9}{2\pi^2})^{1/3}N^{2/3} \quad \text{(S7)}$$

Derivation S3

Bringing equation S1, S2 into Eq. (1) ($\Gamma_{\mathrm{theory}}\sim\lambda N^{\mathrm{a}}R_{\mathrm{g}}^{\mathrm{b}}$), for *N*, we can get:

$$a+b=1 \quad \text{(S8)}$$

Bringing equation S3, S7 into Eq. (1) ($\Gamma_{\mathrm{theory}}\sim\lambda N^{\mathrm{a}}R_{\mathrm{g}}^{\mathrm{b}}$), for N, we can get:

$$a+\frac{1}{3}b=\frac{2}{3} \quad \text{(S9)}$$

Combining equations S8 and S9, we can easily get:

$$a=b=\frac{1}{2} \quad \text{(S10)}$$

Thus, Eq. (1) should be:

$$\Gamma_{\mathrm{theory}}\sim\varphi N^{1/2}{R_{\mathrm{g}}}^{1/2} \quad \text{(S11)}$$

Supplementary Experimental Details

Materials

Acrylonitrile (AN, 99%), poly(ethylene glycol) methyl ether methacrylate (PEGMA, M_n=300), and 2-cyano-2-propyl dodecyl trithiocarbonate (CPDT, 97%) were purchased from Sigma-Aldrich. Dimethyl sulfoxide (DMSO, 99.9%), *N, N*-dimethylformamide (DMF, 99.9%), zinc(II) meso-tetraphenylporphyrine (ZnTPP, 97%), aluminum oxide (Al_2O_3, 90%, basic, 80-160 mesh), perfluoro octane (98%), and hydroxylamine hydrochloride were obtained from J&K Scientific Co., Ltd. Methanol (99.9%) was purchased from Amethyst Chemicals. Potassium hydroxide (KOH, 95%) was obtained from Macklin.

Electrospinning of the BCPs

The BCPs were dissolved in DMF to prepare electrospinning solutions (12 wt.% in DMF) for spinning on an electrospinner (ET-2535H, Beijing Ucalery Technology Development Co., Ltd). A high voltage of 13.5 kV was adopted and the distance between the needle and drum collector was fixed at 20 cm. The diameter of the using needle is 0.51 mm and the flow rate of the solution is 0.52 mL h^{-1} with a constant rotational speed of drum collector at 80 rpm.

Amidoximation reaction of nanofiber membranes

The electrospun nanofiber membranes were treated with 10 *wt*.% hydroxylamine hydrochloride in 50/50 (w/w) water/methanol at 60 ℃ for 4 hours to transform the CN groups into AO groups. The amidoximated membranes were then washed with deionized water 3 times and allowed to dry at 40℃ in an oven overnight.

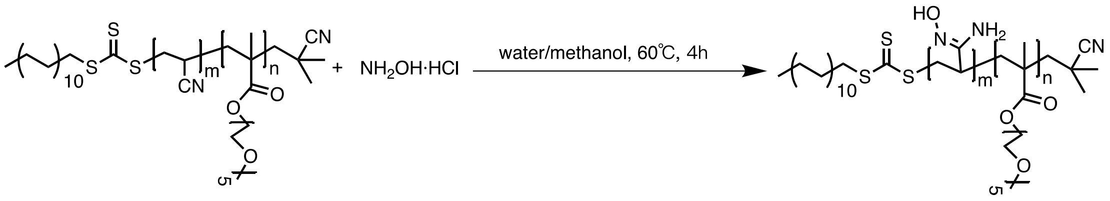

Adsorption tests in uranium-spiked water

Batch adsorption tests of the electrospun nanofiber membranes were first performed in uranium-spiked water. First, 20 μL of uranyl nitrate aqueous solution (188.5 g/L) was mixed into 1 L deionized water. The pH value of the solution was adjusted to be 6.0 ± 0.1 with NaOH. To facilitate the deprotonation of the AO groups in the adsorbents for activating their binding ability toward uranyl ions, potassium hydroxide treatment of the nanofiber membranes was performed at room temperature for 30 minutes in a 2.5 wt.% KOH aqueous solution. After repeated washing, the treated adsorbent was added into 250 mL uranium-spiked water on a rotary shaker (80 rpm) at 25 °C for adsorption experiments. At the time intervals of 5 min, 10 min, 15 min, 30 min, 1 h, 2 h, 4 h, 8 h, 16 h, 24 h, 2 mL of the solution was taken out and analyzed using ICP-OES.

Uranium enrichment in real seawater and elution

A flow-through system was established to evaluate the uranium enrichment ability of the BCP adsorbents in real seawater. Natural seawater was collected from coastal water near the Boundary Island of South China Sea, with an average uranium concentration of 3.3 ± 0.3 ppb. Before delivering to the test system, the seawater was pumped and filtered through a microfiltration membrane (0.22 μm) for removing sediments and microorganisms. The polymeric adsorbent membrane was clamped by two pieces of Basotect melamine resin foam and fixed in the center of a column. All parts that may

contact with seawater in this system were made of non-metal materials to avoid metal ions contamination. The flow rate of the seawater was controlled to be 200 mL/min and the adsorption lasted for 4 weeks. The adsorption capacity of the adsorbents in were analyzed by ICP-MS. After adsorption, the enriched uranium was eluted with 30 mL HCl solution (0.5 mol/L, 10 minutes, room temperature) and the membrane was then regenerated in 20 mL NaOH solution (5 mmol/L, 5 minutes, room temperature).

Characterizations

The molecular weight (M_n) and molecular weight distribution ($Đ$) of polymers were measured by Shimadzu LC-20A GPC system with DMF containing 10 mM LiBr as the eluent at a flow rate of 0.2 mL/min at 40℃ using linear PEO standards for PAN. The dead chain fraction of the polymers was evaluated by a previously reported procedure based on the data processing of the GPC profiles (*Macromolecules*, **2011**, 44, 8028; *J. Am. Soc. Chem.* **2014**, 136, 5508). ^{1}H nuclear magnetic resonance (NMR) spectroscopy measurements were performed on a JEOL ESC-400 (400M Japan) spectrometer. Fourier transform infrared (FT-IR) spectra were obtained by Bruker Vertex 70 equipped with an Attenuated Total Reflectance (ATR) module. Surface morphologies of the nanofiber membranes were recorded by using a Merlin scanning electron microscope (SEM). Inductively coupled plasma-mass spectrometry (for the concentration in ppb level) (ICP-MS) and inductively coupled plasma-optical emission spectrometer (for the concentration in ppm level) (ICP-OES) were used to measure the uranium concentration.

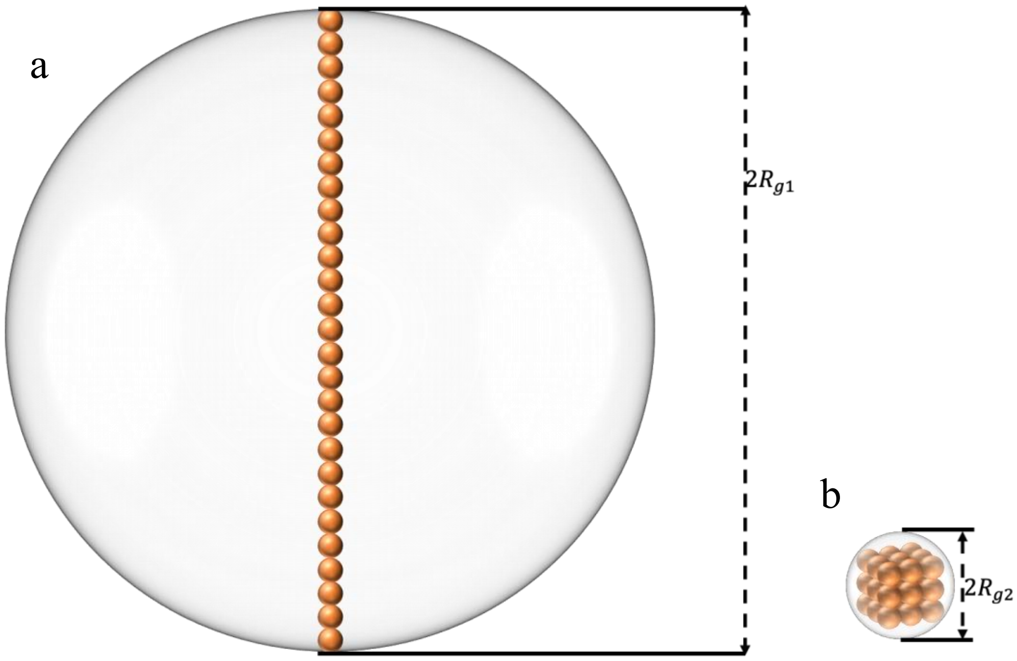

Supplementary Figure 1. Schematic illustration of conceptual polymer chains with fully stretched conformation (a) and completely collapsed conformation (b).

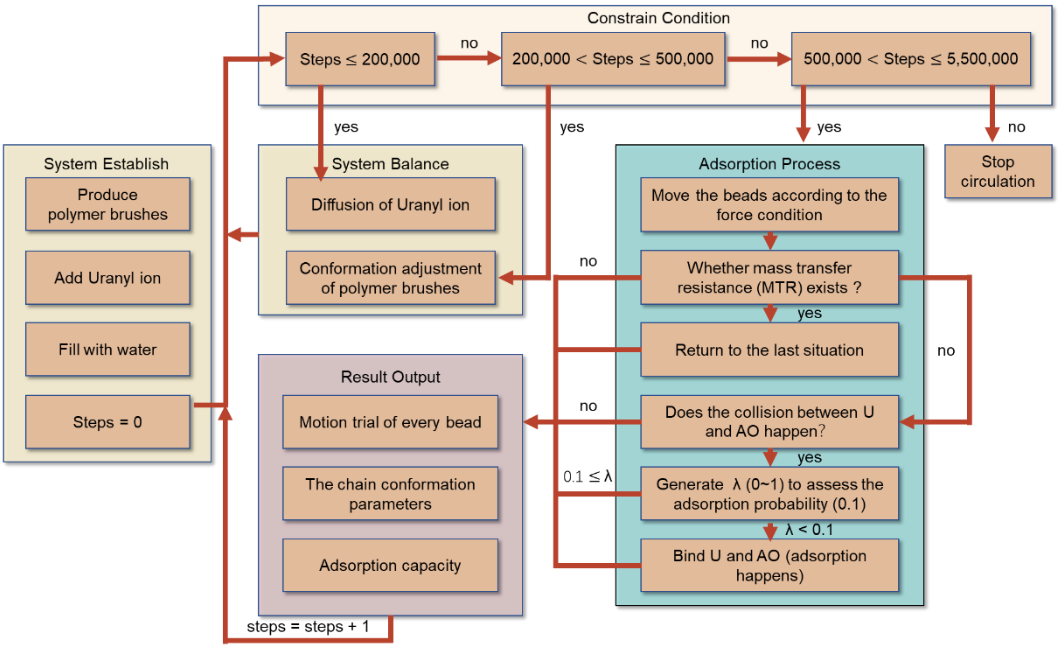

Supplementary Figure 2. A general flowchart implemented in the computational simulation of uranium adsorption by AO-functionalized polymeric adsorbents.

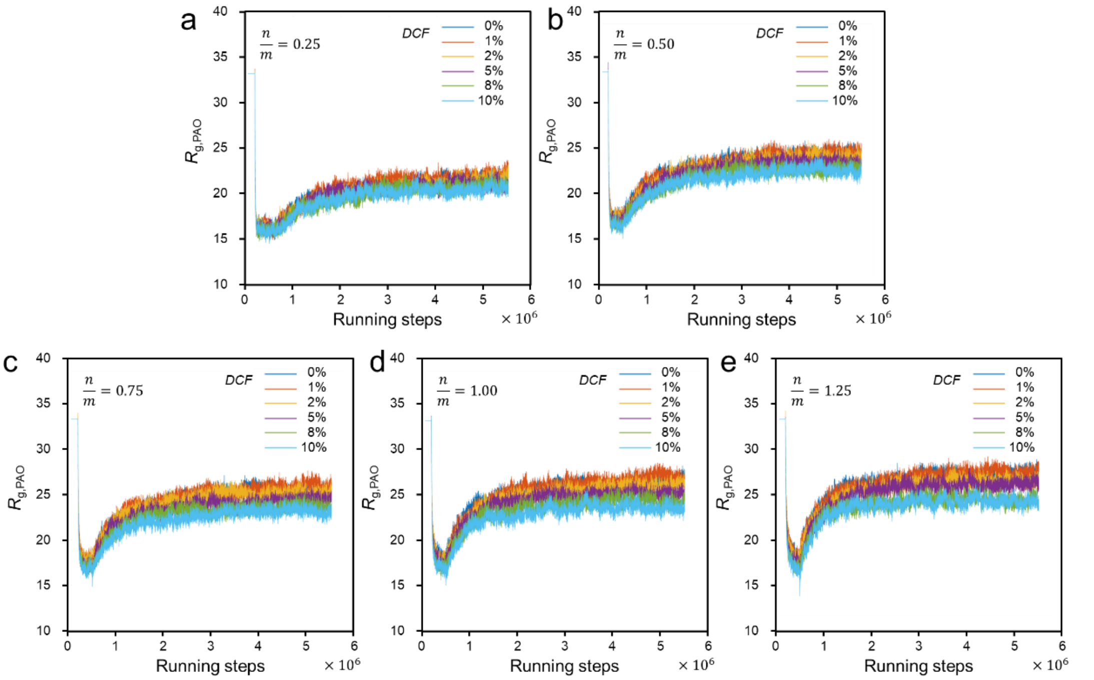

Supplementary Figure 3. Variation of $R_{g,PAO}$ with running steps in computational simulation. **a** PAO_{40}-*b*-$PPEGMA_{10}$ with different DCFs. **b** PAO_{40}-*b*-$PPEGMA_{20}$ with different DCFs. **c** PAO_{40}-*b*-$PPEGMA_{30}$ with different DCFs. **d** PAO_{40}-*b*-$PPEGMA_{40}$ with different DCFs. **e** PAO_{40}-*b*-$PPEGMA_{50}$ with different DCFs.

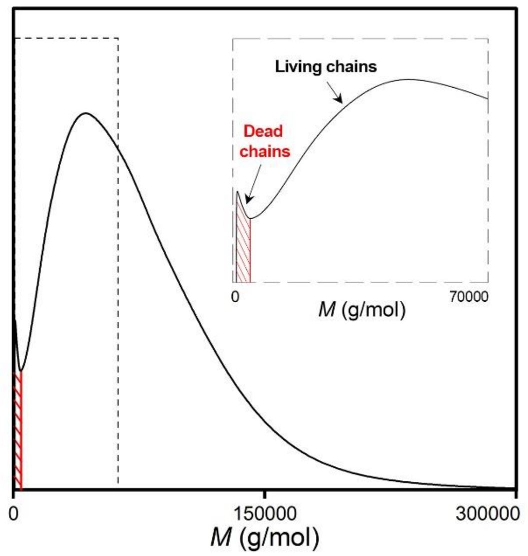

Supplementary Figure 4. Molecular weight distribution (MWD) of the PAN-*b*-PPEGMA obtained in the PET-RAFT polymerization presented as the number distribution. The inset zooms in the red patterned area reflecting the integral domain of the dead polymer chains.

The evaluation method is based on the hypothesis that the low molecular weight tails are attributed to the dead polymers. The MWD is thus converted to the corresponding number distribution for evaluating the dead chain fraction.The red vertical dash line indicates the molecular weight designated as the cut-off point (defined as the minimum point in the number distribution), below which the polymer is assumed to be dead.

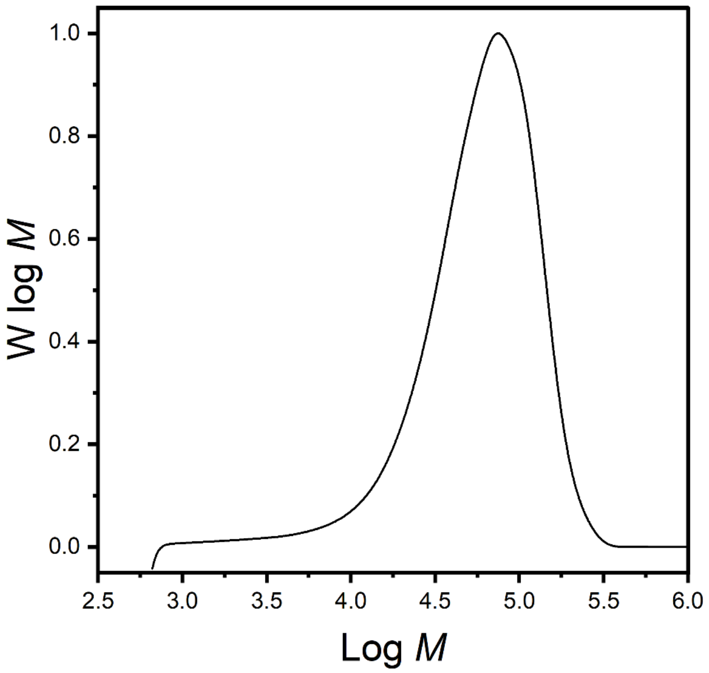

Supplementary Figure 5. GPC profile of the PAN-CTA synthesized in this study.

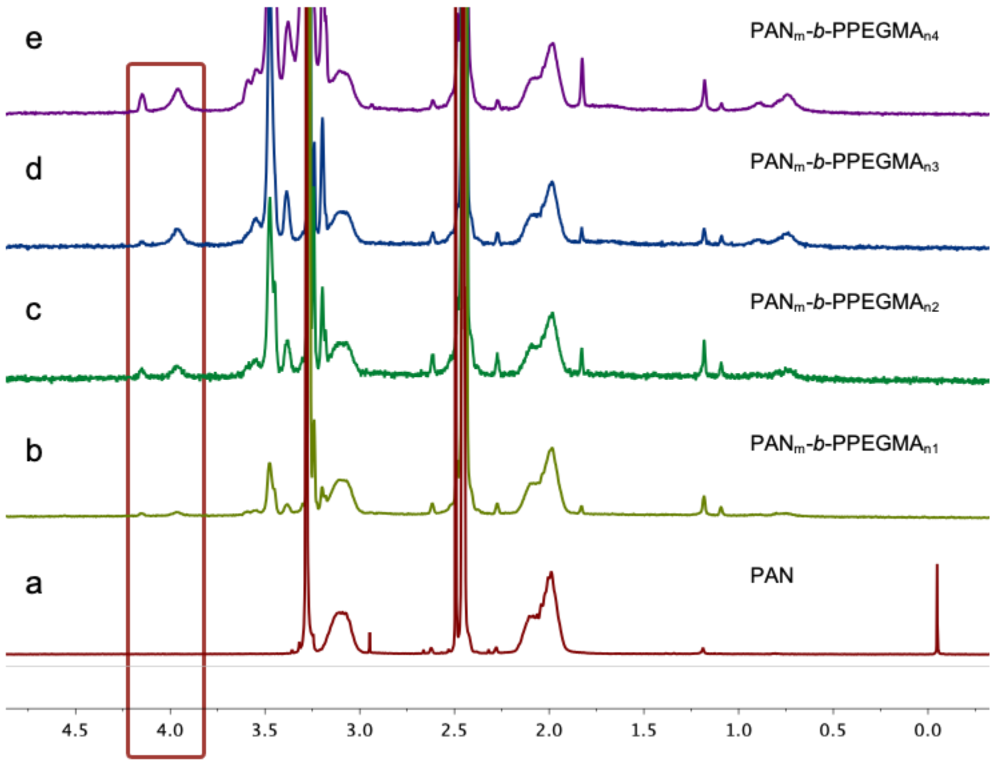

Supplementary Figure 6. ^{1}H NMR spectra of PAN and PAN$_m$-*b*-PPEGMA$_n$ with different block ratios. a Homopolymer PAN. **b** PAN$_m$-*b*-PPEGMA$_{n1}$. **c** PAN$_m$-*b*-PPEGMA$_{n2}$. **d** PAN$_m$-*b*-PPEGMA$_{n3}$. **e** PAN$_m$-*b*-PPEGMA$_{n4}$.

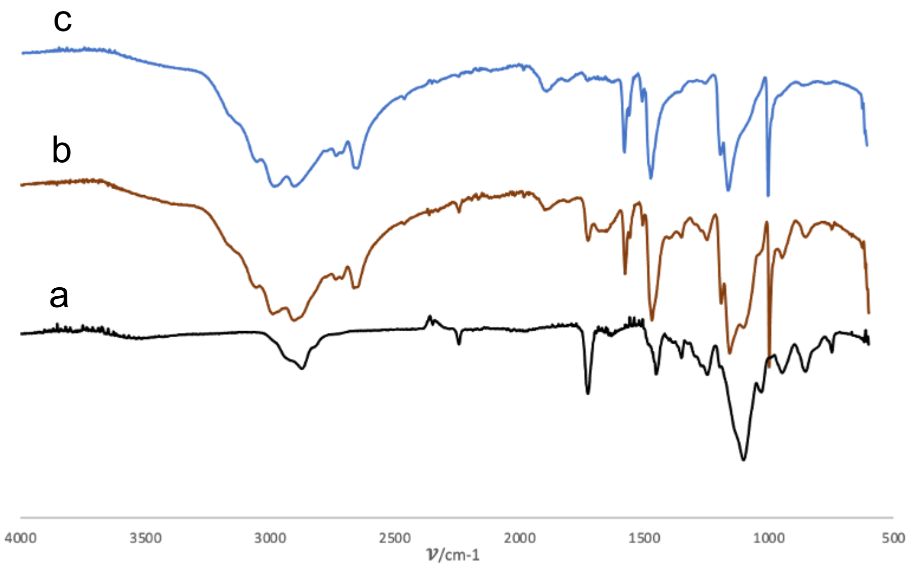

Supplementary Figure 7. FT-IR spectra of homopolymer PAN and the block copolymers. **a** PAN. **b** PAN_m-*b*-$PPEGMA_n$. **c** PAO_m-*b*-$PPEGMA_n$.

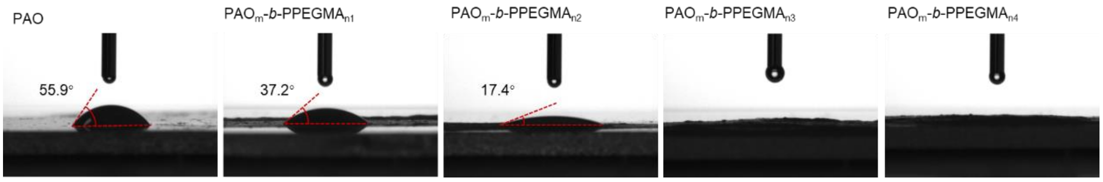

Supplementary Figure 8. Contact angle measurements of the electrospun nanofibrous membranes of pristine PAO and PAO_m-*b*-$PPEGMA_n$ after uranium adsorption. All the membranes exhibited superhydrophilicity after hydroxylamine treatment with the water droplets completely spread out on the surfaces. After exposure to uranium solution, the pristine PAO membrane exhibited significantly enhanced surface hydrophobicity with a contact angle of 55.9 °. In comparison, the PAO_m-*b*-$PPEGMA_n$ membranes, especially those bearing long PPEGMA chains (Table 1), maintained good surface hydrophilicity with small contact angles.

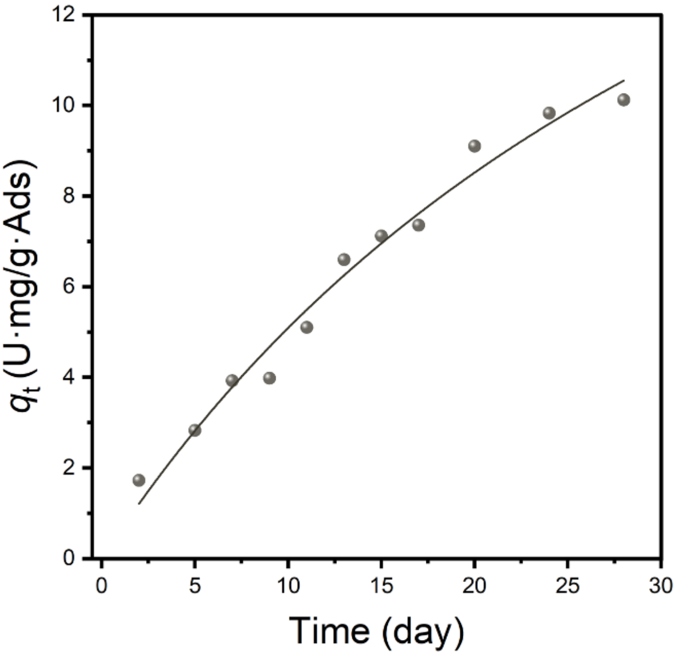

Supplementary Figure 9. Adsorption of uranium in real seawater by PAO_m-*b*-$PPEGMA_n$ with a block ratio n/m of 0.17.

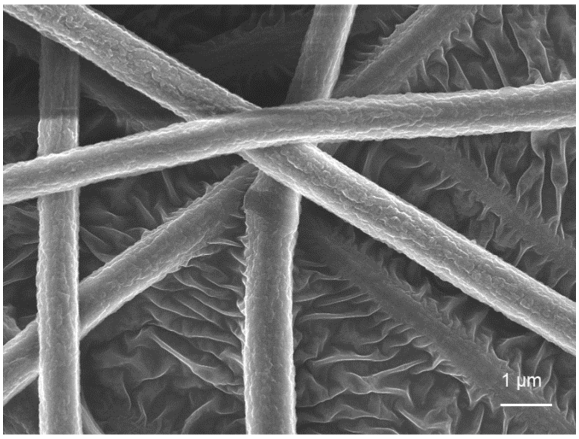

Supplementary Figure 10. SEM image of the regenerated ENM of PAO_m-*b*-$PPEGMA_{n3}$ after uranium elution.

Supplementary Table 1. The linear fitting of the relationship between the equilibrium $R_{g,PAO}$ of PAO_m-*b*-$PPEGMA_n$ with different block ratio n/m and the dead chain fraction (DCF, %) in their structures, $R_{g,PAO} = a \times DCF + b$.

**Block ratio n/m**	***a***	***b***	***R*²**
10/40[a]	null	null	null
20/40	-0.23	24.4	0.946
30/40	-0.27	25.7	0.927
40/40	-0.35	27.1	0.998
50/40	-0.36	27.7	0.939

[a] The linear fitting between equilibrium $R_{g,PAO}$ and DCF for PAO_{40}-*b*-$PPEGMA_{10}$ was invalid due to a quite small R^2.

Supplementary Table 2. The parameters of $\Delta E_{\mathrm{ij}}^{\mathrm{mix}}$ used in the computational study.

$\Delta \boldsymbol{E}_{\mathbf{ij}}^{\mathbf{mix*}}$	**1**	**2**	**3**	**4**	**5**	**6**	**7**	**8**
**1**	0	0	0	0	-0.54	-1.63	-3.44	-3.44
**2**	0	0	0	0	-0.54	-1.63	-3.44	-3.44
**3**	0	0	0	0	0	0	0	0
**4**	0	0	0	0	0	0	0	0
**5**	-0.54	-0.54	0	0	0	-1.99	-0.36	-0.36
**6**	-1.63	-1.63	0	0	-1.99	0	0	0
**7**	-3.44	-3.44	0	0	-0.36	0	0	0
**8**	-3.44	-3.44	0	0	-0.36	0	0	0

[*] i, j=1-8, 1 represents the free uranyl ions, 2 represents the adsorbed uranyl ions, 3 and 4 represent the top and bottom wall beads, 5 represents water, 6 represents PEGMA, 7 represents the active PAO, and 8 represents the dormant PAO.

Supplementary Table 3. The parameters of χ_{ij} used in the computational study.

χ_{ij}*	**1**	**2**	**3**	**4**	**5**	**6**	**7**	**8**
**1**	0	0	0	0	-0.92	-2.75	-5.81	-5.81
**2**	0	0	0	0	-0.92	-2.75	-5.81	-5.81
**3**	0	0	0	0	0	0	0	0
**4**	0	0	0	0	0	0	0	0
**5**	-0.92	-0.92	0	0	0	-3.36	-0.61	-0.61
**6**	-2.75	-2.75	0	0	-3.36	0	0	0
**7**	-5.81	-5.81	0	0	-0.61	0	0	0
**8**	-5.81	-5.81	0	0	-0.61	0	0	0

* i, j=1-8, 1 represents the free uranyl ions, 2 represents the adsorbed uranyl ions, 3 and 4 represent the top and bottom wall beads, 5 represents water, 6 represents PEGMA, 7 represents the active PAO, and 8 represents the dormant PAO.

Supplementary Table 4. Repulsion parameters α_{ij} used in computational study

$\boldsymbol{\alpha_{ij}}$*	**1**	**2**	**3**	**4**	**5**	**6**	**7**	**8**
**1**	25	25	25	25	22	16	6	6
**2**	25	25	25	25	22	16	6	6
**3**	25	25	25	25	25	25	25	25
**4**	25	25	25	25	25	25	25	25
**5**	22	22	25	25	25	14	23	23
**6**	16	16	25	25	14	25	25	25
**7**	6	6	25	25	23	25	25	25
**8**	6	6	25	25	23	25	25	25

* i, j=1-8, 1 represents the free uranyl ions, 2 represents the adsorbed uranyl ions, 3 and 4 represent the top and bottom wall beads, 5 represents water, 6 represents PEGMA, 7 represents the active PAO, and 8 represents the dormant PAO.

Supplementary Table 5. The adsorption capacity and kinetic fitting parameters of the ENMs of PAO_m-*b*-$PPEGMA_n$ in uranium-spiked water.

Adsorbents	Block ratio	Adsorption capacity*	Pseudo-second-order model			Pseudo-first-order model		
			R^2	q_e	k_2 [g mg^{-1} min^{-1}]	R^2	q_e	k_1 [min^{-1}]
PAO	0	182.7	0.961	184.6	8.991×10^{-5}	0.896	166.3	1.17×10^{-2}
PAO_m-*b*-$PPEGMA_{n1}$	0.05	374.8	0.988	387.2	3.424×10^{-5}	0.950	343.2	1.01×10^{-2}
PAO_m-*b*-$PPEGMA_{n2}$	0.08	755.6	0.999	811.0	1.080×10^{-5}	0.980	696.5	7.01×10^{-3}
PAO_m-*b*-$PPEGMA_{n3}$	0.17	958.5	0.999	977.3	3.460×10^{-5}	0.972	878.7	1.37×10^{-2}
PAO_m-*b*-$PPEGMA_{n4}$	0.23	845.2	0.993	840.3	6.256×10^{-5}	0.917	782.7	3.57×10^{-2}

* The adsorption capacity was obtained in 20 ppm uranium-spiked water within 24 h.
